# Advanced Graft Development Approaches for ACL Reconstruction or Regeneration

**DOI:** 10.3390/biomedicines11020507

**Published:** 2023-02-09

**Authors:** Olga Urbanek, Maryla Moczulska-Heljak, Mikołaj Wróbel, Andrzej Mioduszewski, Dorota Kołbuk

**Affiliations:** 1 Institute of Fundamental Technological Research, Polish Academy of Sciences, Adolfa Pawińskiego 5b, 02-106 Warsaw, Poland; 2 Ortopedika—Centre for Specialized Surgery, Modlińska 310/312, 03-152 Warsaw, Poland

**Keywords:** ligament, biomaterial, tissue engineering, regeneration, implant, scaffold, synthetic polymer, natural polymer

## Abstract

The Anterior Cruciate Ligament (ACL) is one of the major knee ligaments, one which is greatly exposed to injuries. According to the British National Health Society, ACL tears represent around 40% of all knee injuries. The number of ACL injuries has increased rapidly over the past ten years, especially in people from 26–30 years of age. We present a brief background in currently used ACL treatment strategies with a description of surgical reconstruction techniques. According to the well-established method, the PubMed database was then analyzed to scaffold preparation methods and materials. The number of publications and clinical trials over the last almost 30 years were analyzed to determine trends in ACL graft development. Finally, we described selected ACL scaffold development publications of engineering, medical, and business interest. The systematic PubMed database analysis indicated a high interest in collagen for the purpose of ACL graft development, an increased interest in hybrid grafts, a numerical balance in the development of biodegradable and nonbiodegradable grafts, and a low number of clinical trials. The investigation of selected publications indicated that only a few suggest a real possibility of creating healthy tissue. At the same time, many of them focus on specific details and fundamental science. Grafts exhibit a wide range of mechanical properties, mostly because of polymer types and graft morphology. Moreover, most of the research ends at the in vitro stage, using non-certificated polymers, thus requiring a long time before the medical device can be placed on the market. In addition to scientific concerns, official regulations limit the immediate introduction of artificial grafts onto the market.

## 1. Introduction

Every year, a large amount of funds is spent on the development of new materials for medical use. Due to restrictive requirements, many problems need to be solved. Anterior Cruciate Ligament (ACL) reconstruction and regeneration is an example of a topic where the capabilities of recently developed materials clash with their limitations.

Ligaments are bundles of connective tissue that connect intra- and extraarticular bones, providing stability to the skeletal system. Examples of ligaments are the anterior and posterior cruciate ligaments of the knee. The ACL is attached to the inner surface of the lateral femoral condyle. It extends obliquely downward in a double bundle or ribbon fashion. In daily and sports activities, ligaments provide anteroposterior and rotational knee stability and are prone to injuries. Injuries occur frequently, and many are eligible for orthopedic treatment [1,2,3,4].

Along with the Posterior Cruciate Ligament (PCL), the ACL is the primary knee stabilizer. A typical sign of ACL deficiency is the feeling of the knee buckling or giving out, especially with a dynamic load. The knee can also be locked in a fixed position, become painful during activities, and swell.

Injuries to ligaments cause an imbalance between the mobility and stability of the joint, leading to an improper transfer of forces through the joint. This can cause damage to the joint’s structures and surrounding tissues. Due to the increasing popularity of sports activities, the number of ligament and tendon injuries is rising every year. As a consequence, the cost of sports injury treatment is also increasing worldwide.

Many risk factors predispose people to ligament tears, including age, lifestyle (active or sedentary), instability of surrounding tissues, chondral problems, osteoarthritis, rheumatoid arthritis, genetic factors, obesity, cartilage tumors, or overuse [5,6,7]. For example, bone tissue (changing with age) plays an essential role in the biomechanical properties of ligaments and tendons because of the development of their attachment to the bone. In addition, patients’ bone density (decreasing with age) affects both ligament reconstruction and physical activity levels before and after surgery. Another significant factor influencing treatment effectiveness is the time from the occurrence of the injury: if too much time has passed, the enzymes present in the synovial fluid degrade the damaged tissue, and as the years go by, the joint’s cartilage frequently deteriorates due to instability. 

Furthermore, according to Credence Research Inc., the market for artificial tendons and ligaments was estimated at $64.3 million in 2021 and is expected to grow at a compound annual growth rate (CAGR) of 10.1% to reach $165.7 million by 2031 [8]. Technological advancements and the rising geriatric population worldwide are some of the key factors fueling this market growth. 

Global reports on the ACL indicate an intensive growth of ACL ruptures. Globally speaking, the highest rate of ACL injury is found in Australia. There, nearly 200,000 primary ACL reconstructions were performed between 2000–2015. From 2000–2015, the annual incidence of ACL reconstructions increased from 54.0 to 77.4 per 100,000. The total cost of ACL reconstruction surgery in 2014–2015 was estimated to be $142 million [9].

According to the British National Health Service report, between 1997 and 2017, a total of 133,270 ACL reconstructions were performed on 124,489 patients, and the group with the highest rate of injuries consisted of women with a mean age of 29.5 years (17%) [10]. Nationally, the age-standardized and sex-standardized rate of ACL injuries increased 12 times from 2/100,000 in 1997–1998 to 24/100,000 in 2016–2017 [10]. There were nearly 300,000 ACL reconstructions in the USA, and the rate of ACL reconstruction per 100,000 people rose by 22%, from 61.4 in 2002 to 74.6 in 2014 [11]. The analysis revealed the highest quality of ACL reconstruction in a group of males aged 13–17 years.

Unfortunately, most commercial products do not provide long-term and successful treatment results, mostly due to insufficient mechanical properties. In the case of artificial grafts, there may not be an optimal proper biocompatibility and/or biodegradation time [5,6]. Researchers are constantly looking for better solutions for ACL reconstruction. So far, in 2022, 440 research articles have been published on “ACL & graft” terms, according to PubMed data. We analyzed a number of publications about various scaffold preparation techniques, e.g., electrospinning, 3D printing, extrusion, etc. A relatively new trend is the formation of hybrid scaffolds, which combine the advantages of different artificial graft-forming techniques. It is worth to mention the clinicaltrials.gov database has 205 records related to ACL injuries (ongoing clinical trials) (accessed on 10 December 2022). Only 35 are focused on various medical devices used in ACL treatment, while only six are related to novel scaffolds for ACL regeneration. It means that only a few solutions analyzed on a laboratory scale reach the clinical trials stage. According to clinicaltrials.gov, ca. 3% of ongoing clinical trials concern novel materials for ACL reconstruction.

This review aims to summarize the current state of the art in ACL treatment methods to determine future trends in advanced graft development. We searched the PubMed database to identify trends for artificial graft development. Possibilities and limitations arising from the available advanced graft formation methods were detected from selected publications. From this information, we prepared a reliable summary of graft-forming processes and applied biomaterials. Moreover, all information was verified and commented on from a practical perspective by materials science engineers and practicing orthopedic surgeons (co-author).

## 2. ACL Morphology and Mechanical Properties

**Figure 1:**
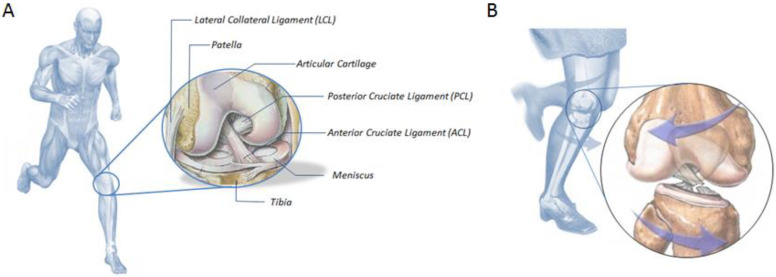
(**A**) Scheme illustrating the ACL location and anatomy. (**B**) An example of knee movement leading to an ACL rupture.

Ligaments are strong connective tissue bands that connect bones intra- and extra-articularly, transferring forces from one bone to another and providing joint stability. The best known are the cruciate ligaments of the knee, further broken down into anterior and posterior. An anterior cruciate ligament (ACL) is among those most prone to injuries. The ACL is a band/tape-like structure of dense connective tissues. It can be broken down into two bundles: anteromedial and posterolateral. It is attached to the inner surface of the lateral femoral condyle and extends obliquely downward, adhering to the tibial anterior intercondylar area (Figure 1A) [12].

In general, its length ranges from 22–41 mm (mean: 32 mm), and its width is from 7–12 mm [13]. The bony attachment size can vary from 11–24 mm across [14]. From its femoral attachment, the ACL runs anteriorly, medially, and distally to the tibia. The ACL’s cross-sectional shape is “irregular” and not circular, elliptical, or any other simple geometric form. Some new anatomical findings, however, describe it as a ribbon-like shape. The cross-sectional area increases from the femur to the tibia, as follows: 34 mm^2^ proximally, 33 mm^2^ mid-proximally, 35 mm^2^ at mid-substance level, 38 mm^2^ mid-distally, and 42 mm^2^ distally [15]. ACL fibers fan out as they approach their tibial attachment, usually in the anterior part of the tibial intercondylar tuberosity. This area is vast and depressed, reaching a width of approximately 11 mm (range 8–12 mm) and a length of 17 mm (range 14–21 mm) in the anteroposterior direction [16]. The distal cross-section is similar to a “C” or “L” shape because it surrounds the lateral meniscus anterior attachment [13].

The mechanical properties of the ACL decrease as patients get older. For the ACL in people aged 22–35 years, the ultimate load was determined to be in the range of 2160 (±157) N. The Young’s modulus for the group aged 22–35 years was determined to be 242 (±28) N/mm [17]. Butler et al. determined the average modulus and ultimate tensile strength by dividing the ACL into regions [14]. The average modulus and ultimate tensile strength measured 278 and 35 MPa, respectively. The ligaments reached their ultimate stress at 15% strain. In a microscale, the ACL consists of three regions, differing in cellular shape, amount of collagen fibers, etc.: the proximal part, which is highly cellular with round and oval cells; the middle part with fusiform and spindle-shaped fibroblasts and a high density of collagen bundles; the distal part with ovoid fibroblasts and a low density of collagen bundles [15]. In most cases, when a knee sprain occurs, the ACL tears in the proximal region, next to its femoral attachment. This section may be divided by subheadings. It should provide a concise and precise description of the experimental results, their interpretation, as well as the experimental conclusions that can be drawn.

## 3. ACL Healing Using Both Classical Methods and a Tissue Engineering Approach

Ligament tears can be divided into three classes, the most serious of which is the complete ligament rupture (Figure 1B). In such a case, either ligament reconstruction with graft or its reattachment to the bone (ligament reinsertion) must be considered. Each group requires a different method of treatment.

Some self-healing processes of ligaments are known, e.g., in the case of medial collateral ligaments (MCL). The bleeding from a ruptured ligament leads to clot formation in a tight envelope between skin and bone, which becomes a natural, artificial graft for migrating cells. Thanks to this, MCL tissue may be restored. Both ACL and MCL ligaments are anatomically very similar, but their self-healing possibilities are very different. The aggressive environment of synovial fluid in the joint is the reason why the ACL does not repair itself like the MCL [18]. It is known that if too much time has passed from the injury, ACL apoptosis occurs, and the self-healing process will not take place. 

Currently, in the case of significant damage to ligaments and tendons, the standard treatment approach is to use autografts [4]. In some cases, autografts are replaced by decellularized allografts or xenografts [8]. The main problems with the auto-, allo-, and xenografts available on the market are shown in Table 1. We do not focus on them in this paper because good reviews are already available [19,20,21]. Table 2 shows the synthetic grafts and membranes/meshes available on the market for ACL reconstruction or regeneration. There are three types of artificial grafts:

- Artificial grafts based on non-resorbable polymers (e.g., Jewel ACL, Lars);
- Artificial non-resorbable tapes used as a bridge for ligament repairs (e.g., Internal Brace, Poly-tape);
- Bio-based and synthetic membranes/meshes used as a bandage for ligament healing.

Tapes are used to reinforce ligament repair procedures, such as stitching the native ligament stump to the femoral attachment. This procedure supports the repaired ligament, preventing its elongation during the healing period without resorbing. The main disadvantage of tapes is that while protecting the repaired ligament, they may also restrict/reduce the ligament’s natural elasticity due to their stiffness.

The list of artificial grafts available on the market is not as long as one might expect (Table 2). Nevertheless, the solutions listed in the table have some limits, e.g., strength, biodegradation time, and reports on inflammatory conditions [22,23,24,25].

An additional problem related to ACL trauma is post-traumatic osteoarthritis, which is most probably associated with an inflammatory response, abnormal joint kinematics, and abnormal stress within the cartilage [26]. Studies have been conducted to evaluate the effect of various ACL treatment methods on further cartilage damage [27,28]. The research conducted on mini-pigs has shown that the most visible cartilage damage appears no earlier than 12 months after the procedure, and some ACL treatment methods may slightly inhibit this process [29]. The aggressive synovial fluid in the joint limits the ingrowth or remodeling of artificial grafts in the same manner as it blocks the self-healing processes mentioned above [28].

Long-term scientific studies show that 7–10 years after primary ACL reconstruction, 20–25% of the patients require revision surgery [2,3,4]. This indicates that despite much scientific and implementation research, there is still a need to develop new, more effective ligament repair strategies.

Numerous scientific publications on the biomechanical aspects of ligaments and tendons have been published in the past decade; however, material and biological ligament issues have not yet been solved.

## 4. ACL Artificial Graft Development in the Tissue Engineering Approach

Gold standard treatments—autograft or allograft reconstructions—or repair procedures do not bring the expected results. For this reason, the overall aim of the research today is to develop a modern, biodegradable graft for reconstructing and regenerating ACLs. Artificial ligaments have been developed by combining materials science, mechanics, biology, and medicine, using different artificial graft formation techniques and their post-treatment.

### 4.1. Essential Requirements of ACL Artificial Grafts

An artificial graft should provide biocompatibility and appropriate mechanical properties and successfully replace natural ligament functions. Artificial grafts for this particular application will have to work in an “aggressive’’ synovial fluid environment. For this reason, their structure should be stable for the time required for cell migration through the graft and should also promote cell activity [31].

Finally, the selection of the most appropriate implantation technique for ACL artificial grafts can be crucial for successful treatment [29]. All surgical implantation procedures should be planned in such a way as to make full use of the advantages of artificial grafts.

### 4.2. Summary of Records from PubMed

We surveyed the literature to evaluate the number of artificial graft fabrication methods and materials from 1 January 1992 to 31 November 2022. Tissue engineering was defined as a science in 1993 [32], so we assumed that from this year on, there would be a rapid increase in implant development, including implants for ACL healing. Data from 1992 was also brought up to check how dynamically tissue engineering in the field of regeneration/reconstruction of ligaments increased. The PubMed database was searched for Titles and Abstracts with the keyword “ligament” and one of the following keywords:

(a) Treatment methods: reconstruction, regeneration, artificial, graft, scaffold, stem cells, growth factors, platelet-rich plasma;
(b) Scaffold forms and fabrication methods: electrospun, extruded, printed, fiber, braid, mesh, sponge, hybrid;
(c) Natural polymers: collagen, gelatin, silk, elastin;
(d) Synthetic polymers: polyester, polyether ether ketone (PEEK), poly (ethylene terephthalate) (PET), polypropylene (PP), polyurethane (PU), poly(lactic-*co*-glycolic acid) (PLGA), poly(L-lactide-*co*-ε-caprolactone) (PLCL), poly(L-lactide) (PLLA), polycaprolactone (PCL).

In all (a–d) groups, the variations of specific words were taken into account: electrospinning/electrospun/electrospin, PCL/polycaprolatone/poly-ε-caprolactone, sponge/sponges, fiber/fiber/fibers/fibers. The total number (T) of publications between 1992–2022 and the number of publications classified as “Clinical Trials” (CT) were shown in graphs (e.g., T: 15; CT:20). The following words were also included: nonwoven, carbon nanotubes, Kevlar bioglass, yarns, nonwovens. However, due to the minimal number of records, they were not shown in the figures (Figure 1A–D).

**Figure 2:**
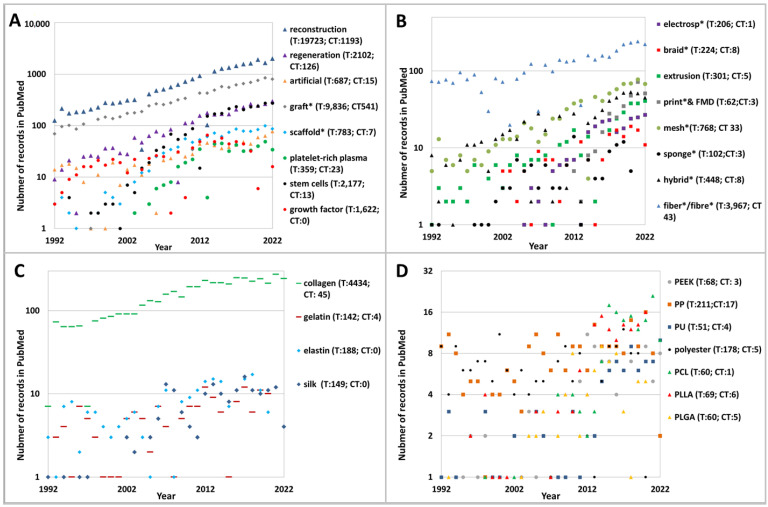
The number of publications in the PubMed database concerned: (**A**) reconstruction, regeneration, artificial, graft, scaffold, stem cells, growth factors platelet-rich plasma; (**B**) electrosp*, extrusion*print*&FDM, fiber*/fibre*, braid*, mesh*, sponge*, hybrid*; (**C**) collagen, gelatin, silk, elastin; (**D**) polyester, PEEK, PET, PP, PU, PLGA, PLCL, PLA PCL.

In scientific literature, there is a growing interest in the reconstruction and regeneration of ligaments (Figure 2A–D). Most scientists lean toward artificial grafts as a suitable form of ACL reconstruction rather than regeneration (Figure 2A). Starting in 2005, much interest was shown in scaffold formation, which proved to be a new standard treatment for tissue engineering (every year, over 10 papers). At the same time, the number of studies on stem cells, growth factors, and platelet-rich plasma as parallel support treatments was also growing. However, in 2015, the number of scaffold development publications began to drop slightly (Figure 2).

Several artificial graft fabrication techniques were analyzed in the scientific literature: electrospinning, extrusion, braiding, and 3D printing. Synthetic grafts were fabricated using fibers, braids, meshes, and sponges. Fibers and meshes were studied, while hybrid, extruded, printed, and electrospun grafts gained attention.

Natural and synthetic biodegradable and nonbiodegradable materials are used: collagen, gelatin, silk, elastin, polyester, PEEK, PET, PP, PU, PLGA, PLCL, and PLA PCL (Figure 2C). Collagen predominated because it is the most present in natural ACLs. There were also more than 10 records per year on silk, elastin, polyester, PCL, and gelatin.

### 4.3. Techniques in Artificial Graft Development

In general, artificial graft properties, e.g., biocompatibility, continuous and uninterrupted pore structure, good mechanical properties, and bioactivity, need to be characterized. Table 3 summarizes the literature on the development of grafts for ACL regeneration.

#### 4.3.1. Fibers

**Figure 3:**
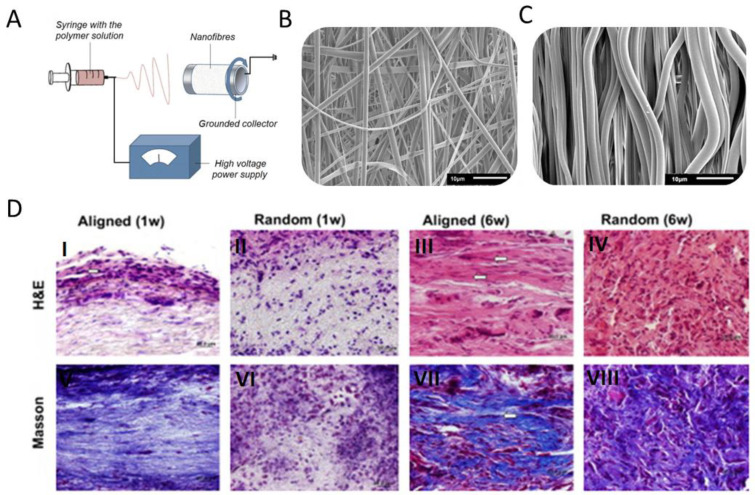
(**A**) Scheme of electrospinning setup; (**B**) SEM image of randomly distributed fibers obtained by electrospinning; (**C**) SEM image of aligned fibers obtained by electrospinning; (**D**) images showing the histological appearance of the cell orientation and matrix arrangement induced by electrospun nanofibers in vivo for 1 and 6 weeks (**I**–**IV**—hematoxylin and eosin staining showing the histological appearance of fibrous scaffold-induced tissue formation; **V**–**VIII**—Masson’s trichrome staining of the bands of collagen fibers) [33]. (Figures cited under the license provided by Elsevier and Copyright Clearance Center, license number 5483771132131).

Electrospinning is the most popular technique for fiber formation. In this technique, the polymer solution jet between a needle connected to a high-voltage supply and a grounded collector is elongated by electrostatic forces (Figure 3A). Electrospinning produces fibers with diameters ranging from dozens of nanometers to a few microns (Figure 3B,C). It is one of the few commercial techniques for nanomaterial formation used on a massive scale [59]. Yin et al. [33] analyzed electrospun nonwoven fibers for ligament regeneration and proved that fiber orientation and artificial graft topography influence gene expression. It was concluded that the aligned fibers lead to a tendon-like tissue formation, while nonoriented fibers lead to osteogenesis and chondrogenesis. Cells cultured on aligned fibers are spindle-shaped, contrary to the round-shaped cells seeded on randomly oriented fibers. In vitro and in vivo results have shown that collagen production is more significant on aligned fibers (Figure 3D). Moreover, aligned fibers had much better mechanical properties for tendon regeneration than randomly oriented fibers [34,60,61]. These results led other scientists to the idea of a dual-drum collector system. This interesting solution enables combining both aligned and random fiber orientation in one material, produced during one single process [35].

Another research group proposed the idea of a nonwoven bundling to fabricate three-dimensional (3D) artificial grafts [36]. Nonwoven bundling significantly increased Young’s modulus and ultimate tensile stress. However, the problem of low scaffold penetration by cells remains unsolved [35]. Barber et al. [62] and Routhrauff et al. [63] conducted similar studies. Barber et al. analyzed mechanical properties and cell response on a 3D scaffold braided from aligned fiber bundles [62]. Their results showed that both mechanical properties and cell response might be achieved depending on the number of braided bundles. Routhrauff et al. [63] compared PCL and PLLA scaffolds in the form of a nonwoven plate and a braided 3D structure (Figure 3E–G). Other researchers suggested nonwovens mimicking the natural tissue composition or proposed to use fibers as a growth factor delivery system [37,64,65]. It was shown that around 55% of bFGF was released in the first week of the experiment. As could have been predicted, cells proliferated faster. In spite of this, collagen production was comparable on both scaffolds, with or without bFGF [64]. It is worth noting that the mechanical stimulation of cells cultured on scaffolds also supports the effects originated from biochemical stimulation and scaffold morphology [66,67]. Plencner et al. [38] have shown that the combination of electrospun fibers with commonly known PP surgical mesh (Prolene) can significantly improve the mechanical properties of the material and cell activity. It has been shown that materials with fibers positively stimulate 3T3 fibroblast cells, leading to their better proliferation and higher collagen synthesis than materials without fibers.

Although many important studies have been conducted on ACL regeneration, there are still numerous fundamental questions concerning, for example, scaffold fixation in the ligament-bone interface. One of the most common ideas is to supplement polymers with hydroxyapatite particles or coat the scaffold surface with this material [39,68,69]. Han et al. proposed an electrospun scaffold prepared using a polycaprolactone, collagen, and hydroxyapatite blend [39]. The nonwoven was wrapped in order to create a 3D structure suitable for the above-mentioned application. The authors presented both in vitro and in vivo results. In vitro studies were conducted for 7 days on an MG63 cell line and confirmed hydroxyapatite’s positive effect on the osteoblast activity and morphology. At the same time, the in vivo tests conducted on rabbits showed that the designed scaffold has significantly better mechanical properties than a pure PCL scaffold within the 8-week period of the experiment. Moreover, new bone was produced between the autologous tendon and bone. This important study confirmed that the idea of a biomimetic approach is highly reasonable [39]. However, there is a lack of long-term studies on rabbits or large animal models that could provide more information about this promising scaffold behavior in in vivo conditions regarding mechanical properties, polymer degradation, or integration with native tissues.

Manning et al. presented a new approach to scaffold design [40]. A layered structure of a heparin/fibrin-based delivery system (HBDS) and PLGA fibers was proposed. This structure was created as a vehicle of selected growth factors and mesenchymal stem cells. This idea made it possible to avoid the issue of the problematic application of hydrogel (HBDS). The authors observed that 22% of growth factors were released within the first day. The remaining amount was released in the following 8 days. However, researchers also observed cell migration from the scaffold [40].

Aligned yarns are another idea for a scaffold for ligament regeneration, which uses the electrospinning technique. Yang et al. proposed the fabrication of a silk fibroin/PLCL nanoyarn formed on a two-collector system, indicating improved mechanical properties [41]. These researchers have proven that this material has a 3D structure characterized by a significantly larger pore size. Moreover, the cells cultured on fabricated yarn show continuous growth over 28 days. This growth was not observed for nonwovens with random or aligned fiber orientation. Yang et al. also revealed a deep scaffold infiltration by cells seeded on nano yarn. However, this effect was not uniform within the sample. As already mentioned in Section 4.1, mechanical stimuli are among the most critical factors affecting tenocyte growth and activity [42].

**Figure 4:**
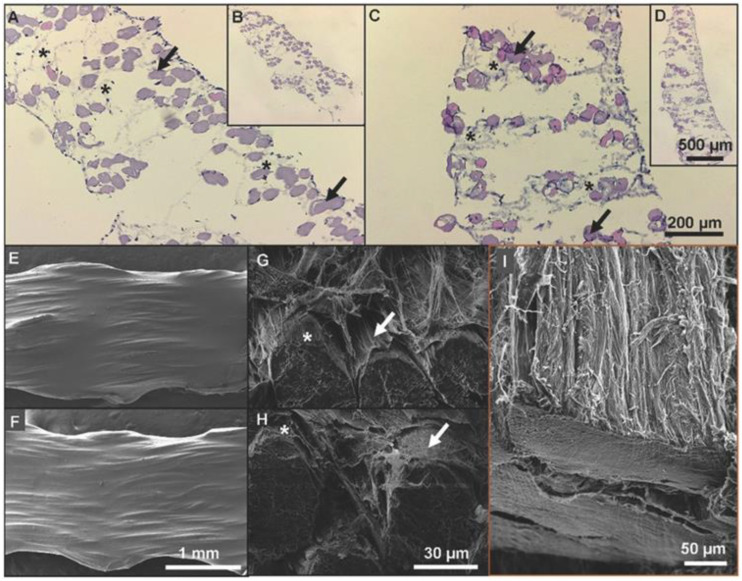
Data obtained by Laranjeira M. et al. [43] presenting cell infiltration and ECM organization on a 3D weave. The histological cross-sections of the scaffolds cultured with cells after 42 days: (**A**,**B**) hTDCs; (**C**,**D**) hASCs (* ECM deposition; → threads stained in pink, while cell nuclei were stained in blue-purple); (**E**–**H**) SEM images show a layer of ECM covering the scaffolds cultured with hTDCs; (**E**,**G**) and hASCs; (**F**,**H**) after 21 days; (**I**) SEM images of a bovine posterior cruciate ligament show a structure similar to both seeded scaffolds. (Figures with the license provided by John Wiley and Sons and Copyright Clearance Center, license number 5483771495280).

Braids of aligned electrospun nanofiber-based on cellulose chitosan and poly-ε-caprolactone were incrementally assembled into 3D hierarchical scaffolds. These 3D scaffolds mimicked the morphology and biomechanical properties of natural ACLs (Figure 4) [43]. The expression of ligament-related markers suggests that the scaffolds developed prevent the phenotypic drift of hTDCs and differentiate hASCs without supplementing biochemical factors. Furthermore, the high porosity permitted cellular infiltration into the scaffolds, which may be a promising feature for the formation of new tissue [70].

#### 4.3.2. 3D Braid

Inter-plaited three-yarn orthogonal sets form 3D braided fabrics, so yarns run through the braid in all three directions. Three-dimensional fabrics can be mass-produced via weaving, knitting, and non-weaving processes [70]. Usually, yarns are formed in the spinning process (melt, dry, or wet) [71,72,73]. The great strength of the scaffold made from 3D braids is achieved thanks to the specific fiber arrangement [74].

Braiding techniques might be used to obtain a 3D structure for ligament tissue regeneration. Up to now, for example, LARS or Leeds-Keio products have been fabricated by this technique. Moreover, one U.S. patent and another relatively new European patent appeared in 2014, based on a new idea of an ACL scaffold made by the braiding process [75]. Essentially, the concept of this scaffold is similar to the Lars Ligament. This scaffold concept, however, was developed by adding some elements, e.g., loops, intraarticular ligament-replacing aspect, or a hollow tunnel [45]. The braiding produces a hierarchical structure of the scaffold, which is very similar to the natural ligament structure. Single fibers form yarns, which are further braided into bundles. By changing the number of braided yarns, braiding angles, or scaffold geometry, the researchers can control selected material properties, e.g., mechanical properties, pore size, and porosity [44].

**Figure 5:**
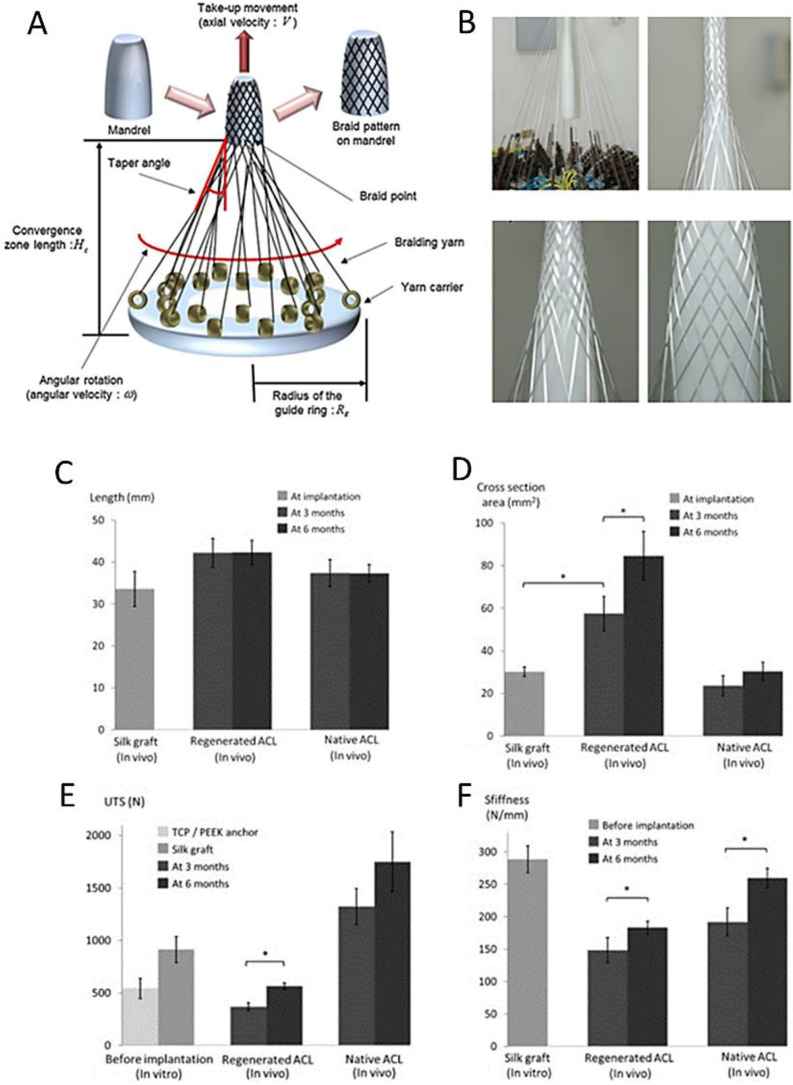
(**A**) Schematic diagram illustrating the braiding process and parameters; (**B**) 3D braids made from single yarns [43] (figures under the license provided by Elsevier and Copyright Clearance Center, license number 5483780312204). (**C**–**F**) Data obtained by Li X. et al. [45] comparing the geometry and mechanical properties of implants at the time of implantation with the regenerated ACL and native ACL at different time points (figures cited under the license provided by John Wiley and Sons and Copyright Clearance Center, license number 5483780489348).

Figure 5A,B show the braiding process and 3D braids made from single yarns. The main paremters of the proces allow to designe required pattern are: the angular speed of the yarn carriers: x; the axial velocity of the mandrel: V; the radius of the guide ring: Rg; the convergence zone length: H c; and mandrel shape [73]. 

Li X. et al. [45] compared the geometry and mechanical properties of implants at the time of implantation with the regenerated ACL and native ACL at different time points. The length and cross-section of: silk graft at implantation, regenerated ligament and native ACL were compered (Figure 4A,B). 

Two regenerated ACL specimens sacrificed at 3 months failed prior to the onset of cyclic loading (with 151 and 184 N, respectively). The ltimate tensile strength (UTS) of the regenerated ACL was 311 ± 103 N at 3 months, and had increased significantly (*p* < 0.01) to 566 ± 29 N at 6 months (Figure 4C). Finally, the stiffness was calculated as the slope of the force–displacement curve between 100 and 250 N of the 250th cycle (Figure 4D). 

Li et al. provided a detailed study on silk fibers (Bombyxmori) enhanced by tricalcium phosphate in a scaffold-bond insertion. Scaffolds were formed on wiring machines: wired, braided, and straight-fibred. Mechanical properties were analyzed in the static and cyclic loading in the dry and wet states. After in vivo studies on pigs, biomechanical tests were performed after euthanasia, with ultimate tensile strengths of 370 N at three months and 566 N at six months, comparable to autograft and allograft performances in this animal model [46]. Tricalcium phosphate proved to be suitable for an enhanced silk graft to the bone attachment. The authors concluded that both initial stability and robust long-term attachment were consistently achieved using the tested structure, supporting an enormous potential for silk–TCP combinations in repairing the torn ACL. It is also worth noting that the developed scaffold is designed to be easily implanted [46]. Some other research papers by Snedeker et al. describe other data on silk-based ACL scaffold development [76,77].

Cooper et al. [44] formed PLGA braids with various yarns and porosities to verify their mechanical properties and cellular response. The resulting braids showed mechanical properties similar to those of the natural ligament. Moreover, the braids had a good porosity and pore size, sufficient for cell infiltration. The same material was further subjected to surface modification by fibronectin to improve cell attachment [47]. Lu et al. [47] have shown that the surface of PGA, PLAGA, and PLLA coated with proteins leads to better cell proliferation and delayed polymer degradation.

Moreover, the presence of fibronectin significantly improved extracellular matrix (ECM) production [48]. Teuschl et al. presented a similar concept of a braided ligament scaffold [49]. They found that braided silk scaffolds provided particular tissue regeneration, albeit not as successful as expected. Silk fiber degraded slower than expected and did not anticipate tissue integration [49]. These results showed that the selected material and scaffold preparation method was not sufficient for successful implantation.

A braided fiber scaffold made of 50% type I collagen (Col-I) and 50% polyvinyl alcohol (PVA) was analyzed for the purpose of anterior cruciate ligament (ACL) reconstruction [50]. An in vivo study was performed on China Bama mini-pigs. Histology and immunohistology analysis showed that the regenerated ligament morphology and major extracellular matrix components resembled the native ACL. Thus, the Col-I/PVA blend fiber ACL scaffold showed good potential for clinical applications [50].

#### 4.3.3. Other Techniques

To achieve appropriate properties, different techniques of scaffold forming and their hybrids are used. In the latter case, the advantages of other methods, such as architecture, pore size, and material type, were combined to achieve the proper scaffold functionality. It is well known that many different factors, such as chemical composition, surface topography, porosity, fiber orientation, and mechanical properties, affect the substrate bioactivity as scaffolding for cells [51].

Liu et al. [52] suggested a 3D braided material combined with a silk-gelatin microsponge as a scaffold for ligament regeneration. The authors stated that in vitro cells showed an increased collagen synthesis in contact with the scaffold. Moreover, four zones (bone, mineralized fibrocartilage, fibrocartilage, ligament) of tissue reconstruction were observed in the in vivo study [52].

Another group of hybrid scaffolds for ACL regeneration is a combination of allografts with synthetic materials. As an example, the study of Smith et al. is described briefly here [53]. For this project, a decellularized porcine diaphragm was combined with nanoparticles of hydroxyapatite and gold in a crosslinking process. This research showed that a ceramic filler influenced the thermal properties of the polymer matrix, which may be important when processing this material. Moreover, depending on the size of the ceramic filler, cell viability was improved. However, no in vivo studies were conducted to prove the osteointegration hypothesis. Mechanical tests should also be performed [53].

Spalazzi et al. [54] published results concerning the multiphase mesh designed for ACL-bone interfaces. The scaffold was prepared using both polymers (polyglactin, poly(lactide-co-glycolide)) and bioactive glass. This research, focused on a topic especially important for a successful ACL reconstruction, showed that a tri-phase scaffold supported heterotrophic interactions, which led to the formation of distinct yet continuous cell and matrix regions. Spalazzi et al. have shown that a proper selection of scaffold formation materials made it possible to obtain high and open porosity and cell interactions in co-culture. Nevertheless, further in vivo studies are necessary [54].

Braided scaffolds were also tested for the bone-tendon interface. A biodegradable and synthetic tri-component graft was produced and evaluated in terms of bone-to-bone healing without the risks associated with harvesting autogenous tissue [55]. It consisted of PLL braids and porous poly(1,8-octanediol-co-citric acid)–hydroxyapatite nanocomposites (POC–HA). After 6 weeks, a histological assessment on rabbits confirmed tissue infiltration throughout the entire scaffold and tissue ingrowth and interlocking within bone tunnels: outcomes that are favorable for graft fixation [56].

A new hybrid structure for ACL regeneration was obtained by electrospinning P3HB or PCL nanofibers and twisting them with silk fibroin (SF) [56]. Two different nano/micro hybrid tissues/structures (SF/ES-PCL and SF/ES-P3HB) were electrospun. The biocompatibility of these scaffolds was confirmed during an in vitro study.

A biphasic sponge scaffold originating from silk fibroin in a freeze-drying process was designed to mimic the gradient in collagen molecule alignment, which is present at the interface [57]. The scaffolds had two different pore alignments: anisotropic on the ligament side and isotropic on the bone side. The researchers proved that this scaffold supported cell attachment and affected cytoskeleton organization depending on pore alignment. In addition, the gene expression of ligament and bone markers significantly changed depending on the pore alignment in each region of the scaffolds [58].

Murray et al. have proven that even by using popular materials such as a collagen sponge and a commercially available bracelet combined with an appropriate implantation technique [29,31,78], it is possible to improve the functionality of the products used separately and obtain great results. A newly developed bridge-enhanced anterior cruciate ligament (ACL) repair (BEAR) method was used. It involves the suture repair of the ligament combined with a bioactive scaffold to bridge the gap between the torn ligament ends. Blood injections and stem cells supported ACL healing. Both in vitro and preclinical animal studies have consistently demonstrated that whole blood is an excellent biological stimulating agent for ACL repair [29,31]. Currently, this solution is under clinical trials.

**Figure 6:**
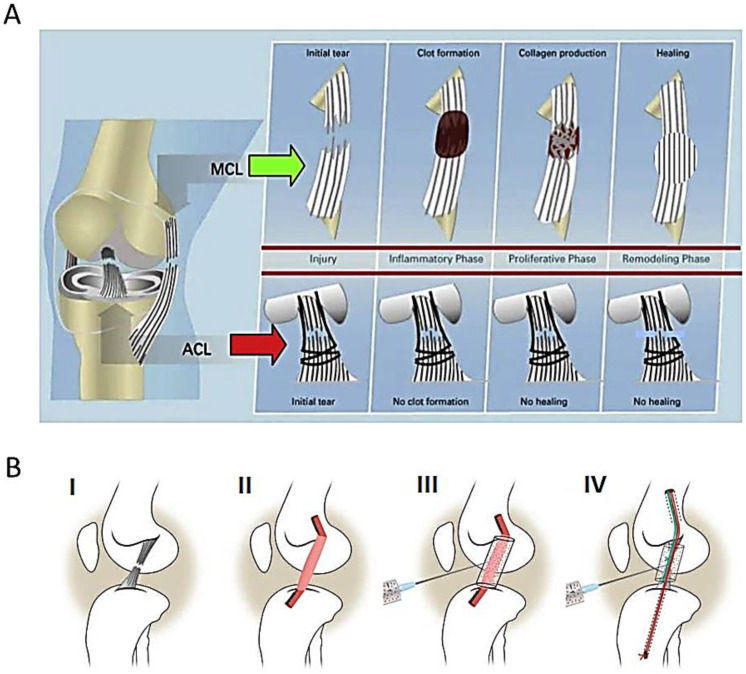
(**A**) New hypothesis of ACL healing failure explained by Murray et al. [78]. After an injury, a fibrin clot is formed on the MCL outside the joint, becoming a natural scaffold for MCL regeneration. At the same time, no fibrin clot occurs on the ACL. Without a clot, healing cannot occur (figures under the license provided by John Wiley and Sons and Copyright Clearance Center, license number 5483780710274). (**B**) Four treatment groups of ACL are shown [29]: (**I**) ACL transection, (**II**) conventional ACL reconstruction, (**III**) bio-enhanced ACL reconstruction around an autograft or allograft to enhance healing, and (**IV**) bio-enhanced ACL repair—bioactive scaffold (extracellular matrix proteins) which activates the platelets in the patient’s blood to release anabolic growth factors, including platelet-derived growth factor (PDGF), fibroblast growth factor-2 (FGF-2), and transforming growth factor-b (TGF-b) into the wound site (figures under the license provided by John Wiley and Sons and Copyright Clearance Center, license number 5483780814636).

New hypothesis of ACL healing failure proposed by Murray et al. [78] is illustrated on Figure 6A. After an ligament injury, a fibrin clot is formed on the MCL outside the joint, becoming a natural scaffold for MCL regeneration. At the same time, no fibrin clot occurs on the ACL. A clot is essential for ligament healing.

Murray at al. [29] presented other treatments used in clinical practice also (Figure 6B): (I) ACL transection, (II) conventional ACL reconstruction, (III) bio-enhanced ACL reconstruction around autograft or allograft to accelerate healing, and (IV) bio-enhanced ACL repair—a bioactive scaffold. The bioactive scaffold, which is based on extracellular matrix proteins found in the normal ACL, activates platelets in the patient’s blood, releasing anabolic growth factors, including platelet-derived growth factor (PDGF), fibroblast growth factor-2 (FGF-2) and transforming growth factor-b (TGF-b) at the wound site.

The most common idea, combining the advantages of ACL grafts and biological strategies, is that of forming hybrids by the medication of the graft surface or structure using growth factors, drugs, or other products to encourage cell proliferation and activity. Growth factors are incorporated into the scaffold structure in other manners also, e.g., plasma treatment combined with further physical adsorption [79], encapsulation, incorporation into fiber structure [80], or incorporation as a hydrogel component [81].

Another approach is the modification of available products: Zhang et al. modified PET from Lars ligament by simvastatin encapsulated in PCL [82]. The authors conducted quite comprehensive in vitro and in vivo studies concerning cell activity after drug release, which proved that the slow release of simvastatin increased the expression of VEGF and BMP-2. This resulted in bone formation on the bone-to-ligament connection on scaffolds with the modified surface.

### 4.4. ACL Scaffold/Graft-Bone Fixation

Currently, one of the most problematic issues in ligament scaffold design is its fixation to the bone. Due to the gradient of ECM composition, mechanical properties, and cell phenotype, it is necessary to design a scaffold that will promote this differentiated part of the tissue [45,83,84]. ACL grafts can be fixed either by screws made from biodegradable polyesters or titanium alloys—so-called interference screws—or by different systems, such as being set on titanium extracortical fixation buttons fixed onto the bone. In this latter instance, the graft is in good contact with the bone inside the bone tunnel. However, the stiffness and strength of fixation are weaker than with screws. Ficek et al. [85] developed PLLA tubes that induce tendon graft osteointegration after ACL reconstruction. Another common idea is to modify the surface of the scaffold, which will also be placed in the bone. Researchers have suggested modifying the surface using calcium phosphate ceramics or bioactive glass [54,57,86]. Moreover, various techniques have been used for this purpose: plasma treatment followed by immersion in simulated body fluid (SBF) [87], incorporation of the ceramic into the fiber bulk [57], or covered by layers of bioactive components encouraging osteointegration [88]. Attempts have also been made to form a gradient structure [36,58]. As expected, most of the suggested solutions positively affected the formation of bone tissue in this area. However, magnetic resonance imagery (MRI) indicates the same limitations of graft-bone fixation, e.g., more lengthy than the declared degradation time in long-term observations, low integration of the screw with the bone, and risk of the screw slipping out. Stolarz et al. [89] summarized bone tunnel enlargement (BTE) phenomena after anterior cruciate ligament reconstruction with interference screw fixation. The authors focused on compiling current knowledge on etiology, diagnosis, and the possibility of reducing this phenomenon occurrence by using the latest methods supporting reconstruction surgery [89].

## 5. Discussion

The aging of the world’s society significantly increases the number of knee injuries. ACL and PCL reconstructions represent the highest percentage of regeneration among all soft tissues, and it is estimated that this group increased by 11.3%, according to CARG, in 2018–2026. The list of the products for ACL regeneration and reconstruction available on the market is relatively short; selected products are used successfully (Table 2). Selected scientific articles report on promising studies on the development of new ACL grafts; however, most of them are in the preclinical stage. As a consequence, orthopedists cannot always guarantee full ACL regeneration.

Summarizing the information on the topic of ACL treatment, we may conclude that:

- There is a significantly strong interest in collagen for the purpose of ACL regeneration;
- The interest in hybrid grafts is growing due to their greater capacity for mimicking graft morphology, mechanical properties, and biological functionality;
- There is a balance between the number of biodegradable and nonbiodegradable scaffolds in scientific literature, suggesting that both approaches to ACL healing are still needed;
- Many publications describe in vitro studies, but only a small percentage of them arrive at the level of clinical trials;
- The development of biodegradable scaffolds for ACL reconstruction is still an unattained goal;
- Increasing attention is being focused on the graft implantation technique, the support of the torn ACL using a sponge scaffold, and the injection of stem cell plasma or the patient’s blood;
- In most cases, scaffold fabrication techniques are easy to upscale to reach mass production, which is an undeniable advantage.

In general, we can see a growing need and interest in the development of ACL synthetic grafts while progress is being made in understanding the biological background of the ACL healing process. Unfortunately, data from scientific literature and researchers’ experience indicate some limitations. First of all, there exists a huge variety of mechanical properties in the grafts developed for the ACL. The ACL graft works in the joint fluid environment, while most data show only dry graft properties. Not all of them meet the requirements for ACL reconstruction. The scaffold should have optimal mechanical properties at the time of implantation and support ligament regeneration through the tissue healing process.

We noted that the biodegradation time of ACL grafts is evaluated in a number of laboratory studies (in vivo or in vitro); many other factors, however, such as the age of the patient or personal metabolism, determine the degradation time in the human body. We found no commercial graft for ACL taking such factors into account for adaptation to individual requirements.

From the orthopedist’s perspective, the final shape of commercially available grafts is protected by patents or know-how of universities or institutions; however, none of the ideas described above can mimic the anatomic ligament shape. An easy implantation of the graft is not taken into account in the studies mentioned. This factor is also crucial in the commercialization process.

In our opinion, another drawback is the fact that most of the grafts are developed using substrates/polymers without GMP in labs not meeting GLP/ ISO 13458 standards. Consequently, most of the researchers’ analyses must be repeated in a GLP/ISO lab, thus generating additional costs and time. Similar problems and results concern the research planning without basic knowledge of low regulation for medical devices (e.g., classification of the product and law regulation concerning this class of products, necessary biocompatibility and safety tests arising from ISO 10993, chemical and process documentation, etc.) and commercialization process. In practice, this leads to the monopolization of the market by any company ready to take a risk before clinical trials.

Finally, the lengthy evaluation of graft effectiveness seems to be a further limitation. New solutions need to comply with numerous legal requirements. Fulfilling them is time-consuming and expensive. In our opinion, while this is appropriate from an ethical standpoint. In the case of complications, the feedback is available only after a few years when recovery is at risk.

## 6. Conclusions and Future Approaches

Restoring an anatomical ACL is still a serious challenge due to its specific morphology, location, and individual patient characteristics. For several decades, we have learned to use synthetic and natural materials in reconstruction and regenerative medicine. There are still two main directions that can be taken in the treatment of torn ACLs. Orthopedists are able to reconstruct ligaments using auto- or allografts or synthetic grafts, or else they may try to repair ligaments using the BEAR method or an internal brace. However, no perfect technique has been developed so far. Therefore, the selection of the surgical procedure is made according to the surgeon’s experience and preferences. This situation becomes seven more complicated in light of new anatomical findings. The idea of changing ligament bundles to a ribbon shape presents unique challenges.

Since the official onset of the tissue engineering sector in 1993 [59], we have believed in the possibility of using biodegradable polymers. However, according to the literature reviewed, biodegradable polymers seem to be a precarious material for ACL restoration. Their mechanical properties do not achieve the strength and resistance requirements for continuous dynamic loads. For this reason, PEEK, PET, and PU grafts have been developed. However, the number of nonbiodegradable grafts indicates that the internal environment of the knee does allow the implantation of materials that perform well in other conditions.

The synthesis of innovative bio-based polymers originating from renewable sources and characterized by a more constant biodegradation time or higher biocompatibility than polyesters or nonbiodegradable polymers seems to be the next step in the road map of ligament scaffold development.

Another approach developed by Murray et al. is ACL healing using collagen sponges (BEAR procedure). A return to regeneration instead of pursuing full reconstruction is gaining the recognition of other orthopedists and scientists.

We live in times when the development of an interdisciplinary field of science, e.g., tissue engineering, regenerative medicine, robotics, genetics, etc., is progressing rapidly. We have the opportunity and responsibility to play a significant role in it. In the end, the effectiveness of the solutions proposed by scientists will be verified by patients.

## Figures and Tables

**Table 1:** Advantages and disadvantages of artificial and natural grafts available on the market.

	Artificial Scaffold	Autograft	Allograft and Xenograft
Disadvantages	- Biodegradation—time of graft longer than declared by manufacturers, which results in poor integration with surrounding tissues and insufficient space for native ECM regeneration - 50% in 7–10 years of natural ligament reconstruction require reoperation - Permanent inflammation in tissues surrounding the implant/scaffold, in the case of the use of certain materials - Poor mechanical properties of artificial ligaments, leading to re-breaking	- Two operations at the same time, which increases the time and cost of the operation and the patient’s recovery time - Reconstruction of large defects requires taking a large amount of tissue from the donor, which weakens the surrounding tissues - Additional scar tissue formed in the donor may limit the mobility of the knee - Lack of autografts that could be used for the reconstruction of ligaments	- Inflammation leading to instability and weakness of muscles around the knee - Low risk of becoming infected with viruses (hepatitis B and C, bovine spongiform encephalopathy) - Poor mechanical properties of grafts due to radiation sterilization - Availability - Price
Advantages	- Variability - Availability - Knee stability - Mechanical properties - Possibility to the top-down design of materials (e.g., chemical composition, structure) - Production for larger scale - Without additional procedure/graft procurement (only cells may be obtained from the patient)	- Without the additional cost of the graft - Without the risk of rejection - Without the risk of becoming infected with viruses (hepatitis B and C, bovine spongiform encephalopathy)	- Variability - Availability - Without loss of the patient’s knee stability

**Table 2:** Bio-based and synthetic polymer-based grafts and patches for ACL reconstruction/repair available on the market (data comes from the official website of these products) [22,23,30]. (* device identifier according to U.S. National Library of Medicine).

Product	Company	Material	Degradation Time	Architecture	Other
Grafts and tapes					
Lars ACL	Corinium Centre (Cirencester, UK)	Polyethylene terephthalate (PET)	Not resorbable	3D braid graft	High tensile strength; The potential for inflammatory reaction
Jewel ACL™ (DI 05060267130495 *)	Neoligaments (Leeds, UK)	Polyethylene terephthalate (PET)	Not resorbable	3D braid graft	Gas plasma treatment surface; 7 mm ID × 710 mm; allowing bone and tissue ingrowth
UCL Internal Brace Implant (DI 00888867220980)	Arthrex Co. (Naples- Florida, USA)	Polyetheretherketone (PEEK)	Not resorbable	3D braid tape facilitation of graft advancement and orientation	-
ACL TightRope with FiberTag (DI 00888867308459)	Arthrex Co. (Naples- Florida, USA)	Polyethylene terephthalate (PET)	Not resorbable	3D braid tape	e.g., ACL TightRope with Fiber Tag, BTB TightRope^®^ Implant
Membranes and meshes					
FlexBand™ Plus (DI 00850003396019)	International Life Sciences DBA Artelon^®^ (Marietta, GA, USA)	Polycaprolactone-based polyurethaneurea	Resorbable	Mesh	As a temporary Support for the healing tissue; used for regeneration, e.g., rotator cuff, Achilles, patellar, biceps, quadriceps
X-Repair (DI B055009)	Synthasome 3030 Bunker Hill Street, (San Diego, CA USA)	Poly-L-lactic acid	Degradable	Mesh	A slowly degradable synthetic mesh
Vicryl Mesh (DI 10705031132719)	Ethicon, Inc. 4221 Richmond Rd., (N.W. Walker, MI USA)	Polyglactin 910 (PLGA)	Resorbable	Woven mesh & knitted mesh	It is coated with polyglactin 370 and calcium stearate to decrease tissue drag and bacterial adherence
Tissue Matrix/Xenograft	Zimmer (La Verne, USA)	Collagen and elastin	Resorbable, integrates with the surrounding tissues	Membrane	Crosslinked

**Table 3:** Summary information on the development of grafts for ACL regeneration.

Technique		Source of the Polymer	Mechanical Properties	In Vitro Studies (Cell Types, Culture Time)	In Vivo Studies (Type of Animal, Size of the Testing Group)	Other Features	Ref.
Electrospun nonwovens		PLLA from Ji’nan Daigang Biomaterial Co., (Jinan, China)	The modulus 22.76 ± 5.63 vs. 0.63 ± 0.56 MPa; *p* < 0.001; stiffness (3.48 ± 1.09 vs. 0.07 ± 0.04 N/mm; *p* < 0.001	analysis of the multidifferentiation potential of the hTSPCs toward osteogenesis, adipogenesis, and chondrogenesis	8–10-week-old female mice, histological examination and Masson trichrome staining	Biodegradable	[33]
		PLGA from Purac Asia Pacific, growth factors from Raybiotech, (Norcross, GA, USA)	Not analyzed	Bone marrow cells from New Zealand rabbits, cultured for 14 days	Not analyzed	Resorbable, with bfgf	[34]
		PLGA and PCL purchased from DURECT Corporation (Cupertino CA, USA)	The tensile strengths of 10–40 MPa; the modulus 190–420 MPa; elongation at break of 60–210%	BMSCS from rats, 3 days culture	Not analyzed	Biodegradable	[35]
		PCL from Sigma-Aldrich (St Louis, MO, USA)	The modulus 1.54 ± 0.26 vs. 14.11 ± 3.76 MPa; ultimate tensile stress 0.45 ± 0.09 vs. 4.74 ± 1.64 MPa;	Tendon fibroblasts from equine superficial digital flexor tendons, cultured for 14 days	Not analyzed	Biodegradable yarn	[36]
		Poly(ester amide)s (PEAs), Bovine serum albumin (BSA) and growth factors from Cedarlane, (Burlington, NC, USA)	Not analyzed	NIH-3T3, 10T1/2, and HCASMC, analysis of viability, metabolic activity and gene expression line line	Not analyzed	Resorbable, FGF added	[37]
		PCL from Sigma Aldrich; Prolene surgical product; growth factors from Sigma Aldrich and Hoffman La-Roche (Basel, Switzerland)	Not analyzed	Mouse 3T3 fibroblast cells; 10 days cell culture	27 Chinchilla rabbits	Resorbable, growth factors incorporated: IGF-1, bfgf, TGF-ß2	[38]
		PCL (Mw = 80 kDa), N,N′-dimethylformamide and calf skin Col type I was obtained from Sigma-Aldrich (St Louis, MO, USA). nHAp produced using a nanoemulsion method.	After 8 weeks of in vivo study: max at failure-60 N, stiffness at failure 16 N	MC3T3-E1 murine preosteoblast cell line; cellular: Morphology, proliferation, and mineralization after 1, 3, 5, and 7 days	24 skeletally mature female New Zealand White rabbits; after 4 and 8 weeks, analyses of histological and biomechanical	Ligament-bone Mesh	[39]
		PLGA from Sigma Aldrich and HBDS	Not analyzed	ASCS; cell culture carried up to 14 days	15 adult mongrel dogs, 9 days of study	Growth factors released from HBDS	[40]
		PLCL from GUNZE Co., *Bombyxmori* silkworm cocoons from Jiaxing Silk Co., Ltd. (Shanghai, China).	The modulus 29.72 ± 1.88 vs. 433.56 ± 48.06 MPa; Tensile strength 3.44 ± 0.21 vs. 39.10 ± 2.89 MPa	Mesenchymal stem cells (MSCs) from Sprague-Dawley rats, cultured for 28 days	Not analyzed	Biodegradable yarn made on a dual-collector system	[41]
		poly(ε-caprolactone) from PuracPurasorb	Yarns sustained 3600 cycles per day for 21 days	Human mesenchymal stem cells derived from bone marrow, cultured for up to 21 days	Not analyzed	Biodegradable yarn	[42]
		Microcrystalline cellulose powder, puri- fied chitosan medium molecular weight, poly-ε-caprolactone (PCL, average M n = 80,000)	Not analyzed	hASCS and hTDCS cells; Alamar Blue test and morphology after 1–28 d	Not analyzed	Electrospun yarns ware used to fabricate 3D braids	[43]
3D Braiding		PLAGA from Ethicon, Somerville, NJ, USA	Maximum load 2.4 ± 0.2 907 ± 132 N; ultimate tensile strength 5.3 ± 1.8 429 ± 84 MPa	Primary ACL fibroblasts from New Zealand White rabbits, cultured 24 h	Not analyzed	Biodegradable	[44]
		Silk fibers (Bombyx mori) (Grege 20/22, Trudel Limited, Zurich, Switzerland); Wiring machine: wired, braided, and Straight fibered	Measured in dry and wet; ultimate Tensile strength 9.4− 7.370.4 N; Stiffness 1.07 −1.47 N/mm. 1.08 In cycling mode, analyzed up to 3% strain at 1 Hz in 250 loading cycles.	Human foreskin fibroblasts (HFFS); braids morphology and mechanical properties were described after cyclic loading	14 healthy adult male pigs (Chinese Tri-hybrid pig: Xianyang breed) aged around 4 months and weighing 55.2 ± 3.7 kg (mean ± SD)	Histological transitions from the silk graft to bone were similar to features of native ACL to bone attachment	[45,46]
		PLAGA, PGA and PLLA from Albany International Co. And fibronectin (Sigma Aldrich)	Tested before and after surface modification by fibronectin	Primary ACL fibroblasts from New Zealand White rabbits, cultured 24 h	Not analyzed	Biodegradable	[47]
		Combination of various non-degradable and degradable polymers, both natural and synthetic	Load strength 500–3200 N, stiffness 200–700 N/mm,	Not described in patent submission	Not described in patent submission	Implanted with seeded cells	[48]
		Bombyx mori silkworm fiber of 20/22 den and 250 T/m (Testex AG, Zurich, Switzerland)	Ultimate tensile strength 1450 N Stiffness 200 N/mm	Not analyzed	33 mountain sheep, 6 and 12 months	Cell seeding led to increased tissue regeneration	[49]
		50% type I collagen (Col-I) and 50% polyvinyl alcohol (PVA)	At 24 weeks, maximum load and tensile strength were 472.43 ± 15.2 N and 29.71 ± 0.96 MPa, respectively	Cytotoxicity on L929 fibroblasts after 4 and 7 days	In vivo on China Bama mini-pigs during 24 weeks	After 24 weeks, morphology and major extracellular matrix components of the regenerated ligament resembled the native ACL	[50]
		Silk	Ultimate tensile strength c.a 1450 N; stiffness 194 N/mm,	In vitro not described in this article	33 female mature mountain sheep	Multi-stage structure of the scaffold—fibers form yarns, and yarns build strands that form the whole scaffold	[51]
Other techniques	Freeze-drying and braiding	Bombyx silk fibers; polymers, e.g., gelatine from Sigma Aldrich	Not analyzed	HMSCS cultured for up to 14 days	48 New Zealand White rabbits, studies carried out up to 24 weeks	Biodegradable	[52]
		Porcine diaphragms were decellularized; AUO and HA nanoparticles	-	L929 murine fibroblast cells from ATCC (Manassas, VA); cytotoxicity, proliferation after 3–10 days	-	Natural porcine diaphragm was harvested from swine euthanized and was used	[53]
	Triphasic component scaffold (melted mesh and microspheres)	Phase A polyglactin 10:90 knitted Mesh (vicrylvkml, Ethicon, Somerville, NJ, USA). Phase B microspheres of poly(D-L-lactide-coglycolide) 85:15 copolymer (PLGA, Cambridge, MA, USA) Phase C microspheres consisting of a 4:1 ratio of PLGA and 45S5 bioactive glass (BG, 20 mm; Mo-Sci Corp., Rolla, MD, USA),	Not analyzed	Primary neonatal bovine (1–7-day-old calves) fibroblasts and osteoblasts for scaffold seeding before implantation	27 male athymic rats (NIH-rnu, 225–250 g, Charles River Laboratories, Wilmington, MA)	Biodegradable scaffold which supports multilineage cellular interactions, infiltration and abundant matrix production in vivo.	[54]
	PLL braid and porous POC-HA	Polylacidpll braids with a (Warwick, RI, USA) and Porous poly(1,8-octanediol-co-citrate)-hydroxypatetytepoc–HA (synthetized in lab)	Maximum load 256.2 N, Modulus of the graft 17.5 MPa,	Not analyzed	Rabbits were euthanized 6 weeks	Biodegradable	[55]
	Silk fibroin-electrospunPCL;silk fibroin-electrospunp3hb	PCL Sigma Aldrich, P3HB from Tainan Biopolymer Company (Taiwan, China), SF silk fibroin Yarns from Kiashahr Co. (Kiashahr, Iran), silk with a linear density of approximately 200 Den	SF/ES-P3HB SF/ES-PCL Maximum load, L_max_ 97.6 ± 11.4 110.5 ± 6.6 respectively; Maximum extension, E_max_ (mm) 9.8 ± 1.4 7.9 ± 1.8 respectively	L929 fibroblasts, Mttcytotocicity after 3 days	Not analyzed	Biodegradable	[56]
	Silk sponge	Bombyx mori silk cocoons from Chul Thai Silk Company (Phetchabun, Thailand) and polyethylene glycol solution (10 kda)	Young modulus 600–1200 kpa	Admscs viability, cytotoxicity and proliferation, and gene expression were determined	-	Biodegradable	[57]
	Sponge of collagen -platelet composite	Bovine tissue in an acidic solution	Range of motion and Lachman tests were done on mini-pigs’ knees after. Days of in-vivo: Preop. Extension (deg) 15.0 ± 4.0–34.4 ± 3.2; Change in flexion (deg) −9.3 ± 3.8–17.0 ± 4.5; Lachman at 15 wks (mm) 1.8 ± 0.9–6.3 ± 0.7	-	21 Yucatan minipigs,	Biodegradable	[58]

## Data Availability

Not applicable.
